# The British Pharmacopoeia

**Published:** 1915-08

**Authors:** 


**The British Pharmacopoeia.** After 16 years, a new edition has appeared, incorporating the Indian and Colonial Addendum of 1900 and official for the whole empire. 43 additions, mainly of modern synthetics, and about 150 deletions have been made. Important alterations in potency are as follows, there being also various minor changes in other Galenicals:

Stronger—Acetum scillae, double strength; Linimentum opii, one-third stronger; Spiritus juniperi, double strength; syrupus chloral, about one-tenth stronger; syrupus codeinae, about one-tenth stronger; tinctura aconiti, double strength; tinctura camphorae compositae, one-tenth stronger; tinctura opii, one-third stronger; tinctura strophanthi, four times stronger; unguentum hydrargyri subchloridi, double strength.

Weaker—Acida nictrici et phosphorici et sulphurici, all made reduced variously one-fourth to two-fifths; emplastrum belladonnae, one-half strength; injectio cocainae dypodermicae, one-half strength; injectio morphinae dypodermicae, one-half strength; liquor hydrargyri perchloridi, one-eighth weaker; pilula phosphori, one-half strength; syrupus ferri iodidi, about two-thirds former strength; tabella trinitrini, each tablet 1-1-30 instead of 1-100 grain; tinctura belladonnae, reduced 3-10 in strength; tinctura colchici, one-half strength; tinctura digitalis, one-fifth weaker; tinctura nucis vomicae, one-half strength; trochiscus acidi carbolici, one-half strength.

The metric system has been adopted, dosage being given in this system first but also in the apothecary's system. Edinburg Medical Journal.

